# Editorial: Model-informed drug development and evidence-based translational pharmacology

**DOI:** 10.3389/fphar.2022.1086551

**Published:** 2022-12-12

**Authors:** Jinxin Zhao, Xiao Zhu, Songwen Tan, Chuanpin Chen, Amal Kaddoumi, Xiu-Li Guo, Yu-Wei Lin, S. Y. Amy Cheung

**Affiliations:** ^1^ Biomedicine Discovery Institute, Infection and Immunity Program and Department of Microbiology, Monash University, Melbourne, VIC, Australia; ^2^ Department of Clinical Pharmacy and Pharmacy Administration, School of Pharmacy, Fudan University, Shanghai, China; ^3^ Xiangya School of Pharmaceutical Sciences, Central South University, Changsha, China; ^4^ Department of Drug Discovery and Development, Harrison College of Pharmacy, Auburn University, Auburn, AL, United States; ^5^ Department of Pharmacology, School of Pharmaceutical Science, Shandong University, Jinan, China; ^6^ Malaya Translational and Clinical Pharmacometrics Group, Faculty of Pharmacy, University of Malaya, Kuala Lumpur, Malaysia; ^7^ Department of Clinical Pharmacy and Pharmacy Practice, Faculty of Pharmacy, University of Malaya, Kuala Lumpur, Malaysia; ^8^ Integrated Drug Development, Certara, NJ, United States

**Keywords:** model informed drug development, pharmacometrics, artificial intelligence, machine learing, translational pharmacology

## 1 Introduction

Model-informed drug development (MIDD) is unarguably the cornerstone of pharmacological research in the 21st century (Marshall et al., 2016; Bi et al., 2019; Lesko, 2021; Rayner et al., 2021; Madabushi et al., 2022). It refers to the strategic creation and integration of mathematical models with throughout plan of execution (key questions, assumption, modelling approach and documentation) to facilitate the decision-making process in pharmaceutical research (Marshall et al., 2016; Madabushi et al., 2022). The applications of MIDD range from novel target identification, formulation design, non-clinical and clinical development and biopharmaceutical research to trial design and cost-effectiveness evaluations (Madabushi et al., 2022). MIDD is also increasingly used to evaluate causal links between drug physiochemical properties, disease/pathogen biology and patient physiology. This has facilitated an integrated approach for effective trial designs and data-to-knowledge transformation while also helping narrow knowledge gaps and maximise the therapeutic potential of drug candidates (Wang et al., 2008; Lesko, 2021; Madabushi et al., 2022).

This multifaceted tool has revolutionised the scope of recent drug discovery and development efforts. Over the last decade, the application of MIDD methodologies has become a popular approach for drug development and applied pharmacotherapy projects in industry, academia and, more notably, in global regulatory agencies (e.g., FDA, EMA, ICH, PMDA and NMPA) (Sato et al., 2017; Li et al., 2019; Marshall et al., 2019; Madabushi et al., 2022). At least 90% of all US FDA-approved pharmacological agents have an MIDD-based research origin (Madabushi et al., 2022). Most recently, its applications have been crucial for rapid vaccine and treatment development against the COVID-19 pandemic (Xiong et al., 2022).

This research topic aims to bring together scientists from academia, industry, and regulatory authorities to discuss the most recent advancements in MIDD and their multidimensional role in drug discovery and development. This editorial seeks to discuss current research trends in the field of MIDD and translational pharmacology and share our perspective on the importance of MIDD in modern drug discovery and development.

## 2 Application of model-informed drug development (MIDD)

There is a long-standing history of applying MIDD to inform regulatory decisions at the FDA (Madabushi et al., 2022). In the early 90s, the utilities of MIDD were limited and focused only on product characterisation. The scope of MIDD applications rapidly expanded in the first decade of the 21st century. Since 2013, the FDA has published numerous regulatory guidelines on MIDD, which include characterising safety, guiding trial design, guiding dose selection and assisting effectiveness evaluation (Madabushi et al., 2022). The applications of MIDD are continuously evolving and, in the late 21st century, physiologically based pharmacokinetic (PBPK) modelling and simulations have become prevalent, in addition to classical pharmacometrics applications in modern drug development (Madabushi et al., 2022). In the modern era, the rise of novel mechanistic-based methodologies, such as quantitative systems pharmacology (QSP), is another significant development (Bradshaw et al., 2019).

## 3 Utilities of model-informed drug development (MIDD)

The rampant increase in MIDD-related publications over the last few years is a testament to the piqued interest in the scientific community. MIDD techniques enable the integration of data from clinical trials and non-clinical investigations in a drug development programme. Modelling techniques that are frequently employed include population pharmacokinetics (popPK) modelling, PBPK modelling and exposure-response modelling (Marshall et al., 2016; Keizer et al., 2018; Bi et al., 2019; Marshall et al., 2019; Darwich et al., 2021; Lesko, 2021; Madabushi et al., 2022). Currently, several stages in the development of new drugs have also made use of emerging modelling techniques, such as QSP modelling and artificial intelligence (AI)/machine learning (ML) (Madabushi et al., 2022). Drug development is complex, and a combination of different modelling approaches must often be utilised to adequately address questions that arise at various stages.

Pharmacometrics is the core of MIDD and is the science of developing mathematical models to quantitatively describe relationship exposure and response (Madabushi et al., 2022). In the absence of exposure, kinetic–pharmacodynamic (K-PD) models are used to predict the time course and magnitude of drug effects (Ooi et al., 2020). Kang et al. successfully demonstrated the utility of a K-PD model in describing the anticancer effect of BoNT/A1 in a syngeneic mouse model transplanted with melanoma cells (B16-F10). The developed K-PD model adequately captured the dynamics of tumour growth, and simulation studies were conducted to determine the optimal dose under various dosing scenarios.

Pharmacometrics is not only applicable to oncology but is also widely utilised in anti-infective programmes (Rayner et al., 2021). Mathematical modelling has been employed for the last decade to optimise dosing regimens for antibiotic therapy against multidrug-resistant ‘superbugs’ (Bulman et al., 2022; Yow et al.). These models include PopPK models, QSP, system-based models, and mechanism-based PK/PD models (Rayner et al., 2021). With antibiotics, inappropriate dosing may result in therapeutic failure or toxicities. Precision dosing, which customises doses to individual patients, is crucial for antibiotics, especially those with a narrow therapeutic index. Treatment response in individuals for antibiotics depends on three determinants: the patient, bacterium and antibiotic (Wicha et al., 2021). These factors determine the optimal dose of an antibiotic to maximise efficacy and minimise toxicity and the emergence of resistance. For some antibiotics, treatment responses vary greatly between individuals due to genotype, disease characteristics and patient population. Variability in individual responses to antibiotics demands precision dosing. The traditional ‘one dose fits all’ does not consider these variabilities; hence, therapy for patients may be suboptimal (Yow et al.). Particularly in the case of special populations, therapeutic drug monitoring (TDM) is often used to ensure that the exposure of the drug is optimal by comparing plasma concentration levels against a therapeutic range (Smith et al., 2021; Wicha et al., 2021). Based on this, recommendations are made that often involve dose adjustments to optimise outcomes. Several limitations are associated with the traditional TDM approach—the most significant often relying on trough samples, which is a suboptimal surrogate of overall drug exposure (Wicha et al., 2021). Model-informed precision dosing (MIPD) utilises pharmacometrics principles to integrate various sources of information to streamline the TDM process and maximise therapeutic success (Keizer et al., 2018; Wicha et al., 2021; Bulman et al., 2022). In MIPD, the measured drug concentrations from TDM are used to derive the individual PK parameters that account for the interindividual differences using Bayesian estimation (Smith et al., 2021). These individual PK parameters are specific to the patient of interest and can be used to derive a personalised dosing regimen. Numerous techniques have been proposed for MIPD, including model-averaging (Uster et al., 2021) and hybrid ML/PK approach (Hughes and Keizer, 2021). In this reach topic, Yow et al. presented an excellent overview of various strategies for optimising antimicrobial therapy and the urgent need for implementation of MIPD for antibiotics in clinics.

Unlike the traditional pharmacometric models, which rely largely on available preclinical or clinical PK data and model structures that are selected based on statistical methods and biological plausibility grounds, QSP is a quantitative approach that incorporates mechanistic information of a biological system and drug mechanism to predict and define disease pathophysiology and therapeutic interventions (Sorger et al., 2011; Bradshaw et al., 2019; Helmlinger et al., 2019; Azer et al., 2021; Aghamiri et al., 2022). QSP models can be utilised throughout various stages of drug development (Bradshaw et al., 2019). In the early drug discovery stage, QSP models can be used to identify novel targets. QSP models can be applied in the translational stage to bridge non-clinical to clinical work and are subsequently used to study the source of variability in response in the clinical development stage. Rieger et al. implemented a novel QSP model of human hepatocyte lipid metabolism and demonstrated the suitability of the model in generating a virtual population that closely resembles patients with non-alcoholic fatty liver disease. The treatment intervention was also validated by simulating pioglitazone and diet intervention in the virtual population. As outlined by the authors, the benefits of the QSP model lie in its size and speed, which enables the simulations of large virtual patient populations for hypothesis testing to respond to critical drug development questions in a timely manner (Rieger et al.). Currently, QSP modelling to inform key decisions in drug development is still evolving, and only a handful of successful examples are available in the literature (Sorger et al., 2011; Bradshaw et al., 2019; Helmlinger et al., 2019; Aghamiri et al., 2022). As QSP continues to evolve, the acceptability of QSP for external and internal decision making will undoubtedly increase.

Lastly, the use of AI and ML has been increasing in the pharmaceutical industry to overcome the high failure rate in drug development (Zhang et al., 2017; Koromina et al., 2019; Saikin et al., 2019; Liu et al., 2020; Talevi et al., 2020; Gupta et al., 2021; Kashyap and Siddiqi, 2021). As such, the industry is beginning to explore and implement various AI and ML frameworks to supplement or be integrated into current drug discovery and development processes (Liu et al., 2020; Talevi et al., 2020). Yu et al. developed and demonstrated the utility of a supervised machine learning model to categorise and examine the magnetic resonance imaging features of brain tumours. Implementation of AI/MI in drug discovery and development will assist with the interpretation of clinical data and can standardise results across labs, thereby reducing biases and human errors (Zhang et al., 2017; Koromina et al., 2019; Saikin et al., 2019; Liu et al., 2020; Talevi et al., 2020; Gupta et al., 2021; Kashyap and Siddiqi, 2021). The future prospect of the utility of AI/MI in drug discovery and development remains unclear. There is an urgent need to bring together scientists from academia, industry, and regulatory authorities to outline critical research priorities and work towards best practices with respect to the use of AI/MI and to resolve any regulatory hurdles associated with the use of AI/MI in drug development. The development of standards for AI/MI and the implementation of best practices will undoubtedly boost confidence in the community in adopting AI/MI to facilitate and aid drug discovery and development (Liu et al., 2020).

## 4 Conclusion

MIDD has developed into an effective method to aid modern drug discovery and development. It plays a critical role in regulatory decision making and is gaining more acceptance in the community. Incorporating and leveraging newer techniques, such as MI/AI, are on the rise and will undoubtedly reshape drug development in the long term.
